# When Technology Exceeds its Application: The First Reported Reconstruction of the Iliocaval Confluence Using an Aortic Endograft

**DOI:** 10.7759/cureus.4640

**Published:** 2019-05-10

**Authors:** Taylor S Harmon, Victoria V Villescas, Preston Hood, Travis E Meyer, Jerry Matteo

**Affiliations:** 1 Radiology, University of Florida College of Medicine, Jacksonville, USA; 2 Diagnostic Radiology, University of Florida College of Medicine, Jacksonville, USA; 3 Interventional Radiology, University of Florida College of Medicine, Jacksonville, USA

**Keywords:** deep venous thrombosis, venoplasty, venous insufficiency, aortic endograft, iliocaval reconstruction, post-thrombotic syndrome, pulmonary embolus, iliocaval confluence, chronic venous insufficiency, interventional radiology

## Abstract

Severe venous dysfunction in the setting of subacute iliocaval occlusion is a high cause of morbidity and mortality in patients. Fortunately, the development of the appropriate interventional management has allowed for better patient prognosis, despite device limitations. Severe cases of venous insufficiency, anatomically challenging vasculature, and device failure remain imperative when discussing the caveats for interventional success. The current gold standard of treatment for iliocaval disease has proven to be venoplasty in conjunction with stent placement within thrombotic occlusive areas. Though intuitive for modern day interventionists, this standard is not always forthright, especially when the most prevailing interventions fail to adequately treat certain venous pathologies. In this case, interventional operators must be willing to adapt their technical proficiency and knowledge of readily available devices to successfully treat the progressive nature of venous insufficiency. The following report demonstrates an example of how an interventional operator acclimated their interventional approach to successfully treat a severe and technically challenging case of subacute iliocaval occlusion, using an aortic endograft. In this first documented deployment of an aortic endograft in an iliocaval confluence, the results show resolution of the patient’s subacute iliocaval occlusive disease, as well as complete iliocaval patency and the absence of post-procedural complications.

## Introduction

Chronic and acute-on-chronic iliocaval disease has been historically difficult to manage, based on the limited treatment options that have been available. Both structural and functional venous insufficiency has often been undertreated medically, which serve to only resolve acute venous etiologies, but do not address post-thrombotic venous disease. In 1984, Plate et al. first recognized the poor prognosis for patients who developed post-thrombotic disease as a result of anticoagulation management [1-2]; 59% of those documented patients who were managed with anticoagulation developed severe thrombosis in their lower venous systems, due to thrombotic lesions in the follow-up period [2]. At that time, the only alternative to medical management for patients who continued to have post-thrombotic disease was surgical intervention, proven to be an inefficient means for treatment [1,3].

In all published studies of surgical interventions for both acute venous and post-thrombotic disease, the largest patient cohort is ten, with a reported venous patency of 45% to 85% [1,3]. In fact, most documented literature attributes venous reconstruction suitable for venous trauma, rather than acute or chronic venous disease [4]. It was not until the early 1990s when percutaneous management was introduced as a valid alternative to medical and surgical management.

Mechanical saline jet thrombectomy and balloon venoplasty were first described by Yamauchi et al. in the setting or treatment for acute thrombosis of the inferior vena cava (IVC) [5]. The same report described the first deployment of bare metal-balloon expandable stents in the IVC to maintain patency. Since then, many documented reports have described a combination of venoplasty and bare metal-balloon expandable stent placement as the gold standard of treatment for iliocaval disease [1,6-8].

Presently, venoplasty and uncovered bare metal-balloon expandable stent placement for the maintenance of venous patency continues to be the gold standard for iliocaval disease. Especially in the setting of chronic iliocaval disease, the proper clinical work-up and conjunctive imaging studies can assist in the decision to pursue interventional management of chronic venous dysfunction. However, there are caveats to the currently accepted interventional management of venous disease. One is the extent of venous thrombotic damage, resulting in the inadequate endovenous interventional management if severe. In this situation, the amount of thrombosis cannot be resolved by venoplasty. Another is the area of thrombotic damage. For example, endovenous stenting within the iliocaval confluence has been reported to cause contralateral iliac vein and post-procedural deep vein thrombosis [9]. However, both challenging anatomical areas, such as the iliocaval confluence, and extensive thrombotic occlusion can be addressed when considering venoplasty and venous reconstruction in chronic or acute iliocaval disease, according to recently documented novel interventional approaches.

In 2018, Matteo et al. described a novel method for repairing the iliocaval confluence in a trauma patient requiring covered kissing stents [10]. In this documented case, the diameter of iliac stents needed to accurately repair the venous lumens were solved based on the diameter of the native inferior vena cava. The post-procedural outcome showed no apparent post-procedural complications such as venous thrombosis or endoleaks. In patients that have chronic or acute-on-chronic iliocaval disease at the iliocaval confluence, the “Matteo mathematics method,” utilizing covered kissing stents, might be an appropriate option. However, if the patient has extensive thrombotic damage that extends far past the confluence, this method might be limiting. The following case demonstrates a novel interventional method for repairing extensive and severe iliocaval thrombosis at the iliocaval confluence, in a patient with subacute-on-chronic venous insufficiency, using an aortic endograft.

## Technical report

A 51-year-old male presented with intermittent chest pain for two weeks, worsening with ambulation and activity. The patient underwent computed tomography angiography (CTA), which demonstrated a right lower lobe pulmonary embolism.

Thrombolysis of pulmonary embolus

**Figure 1:**
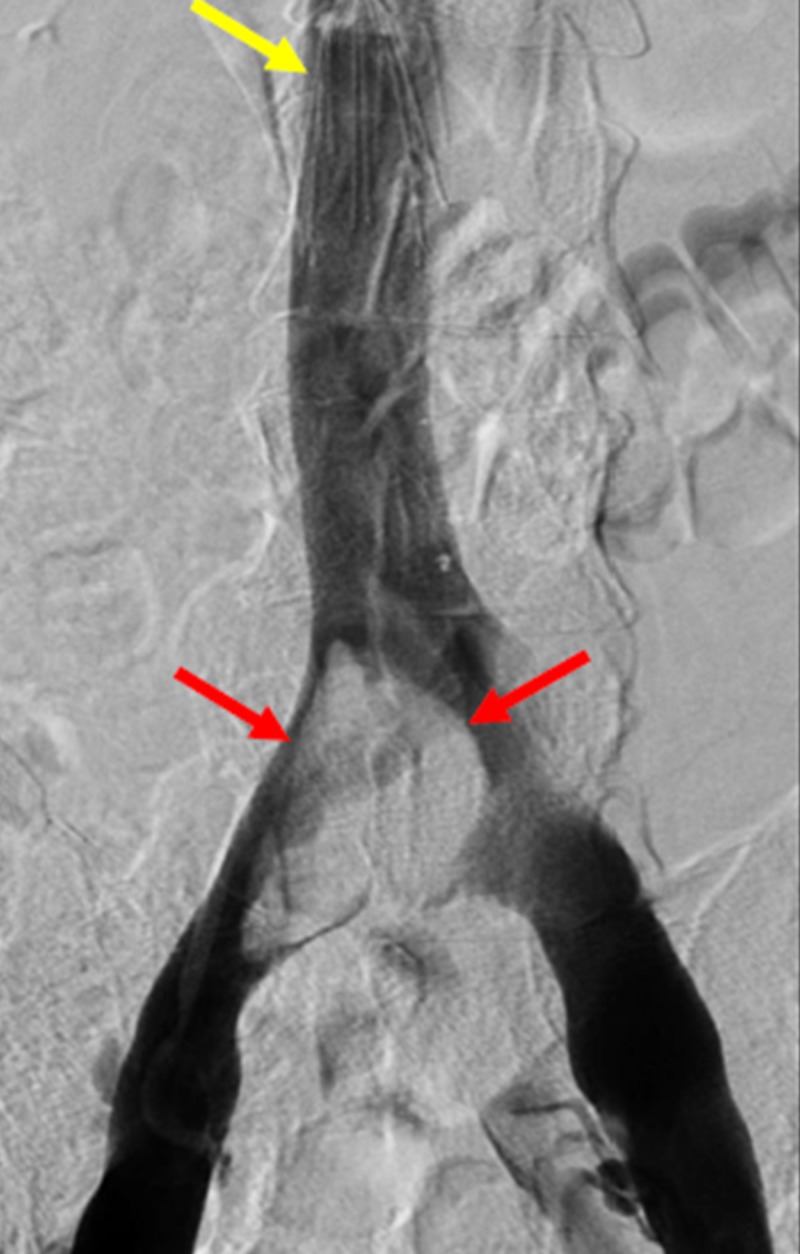
Pelvic Angiogram Demonstrating Extensive Thrombus within the Iliocaval Confluence A pelvic angiogram demonstrates a large filling defect (red arrows) within the region of the confluence of the inferior vena cava, representing thrombus extending into the right iliac vein. Additionally, there is an infrarenal retrievable inferior vena cava filter (yellow arrow).

An emergent pulmonary thrombolysis using the EkoSonic® Endovascular System (EKOS) catheter (EKOS Corporation, Bothell, Washington, US) was performed. An IVC filter was placed at the time of initial thrombectomy. The EKOS catheter was removed from the right groin sheath and exchange was made for a pigtail flush catheter. A right pulmonary angiogram was performed which demonstrated significant improvement in clot burden of the right lower lobe pulmonary artery. The pigtail catheter was then removed and venacavogram was performed via the bilateral groin sheaths, which demonstrated a large thrombus at the confluence extending into the right common iliac vein (Figure 1).

Venoplasty of the iliocaval confluence

**Figure 2:**
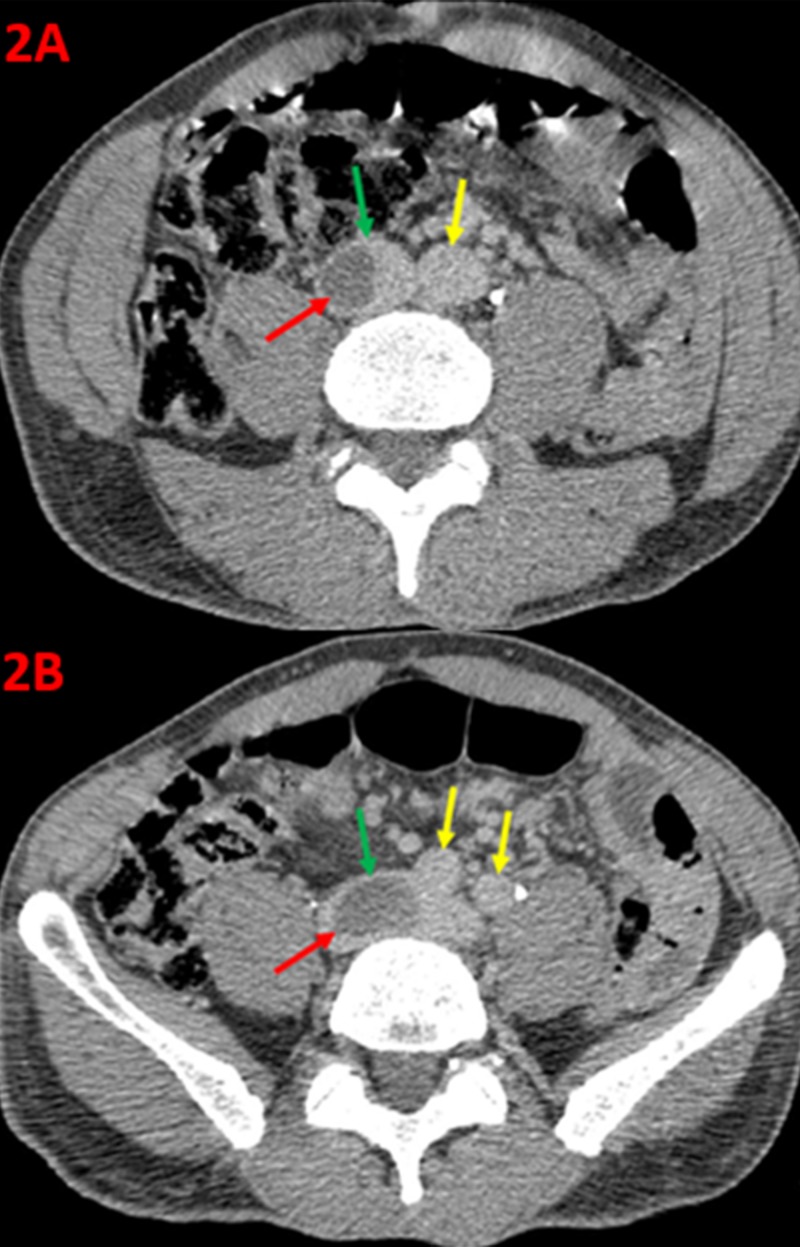
Axial Computed Tomography of the Abdomen and Pelvis An axial contrast-enhanced computed tomography of the lower abdomen (2A) demonstrates the aorta (yellow arrow), the inferior vena cava (green arrow), and a filling defect (red arrow) within the inferior vena cava, consistent with a thrombus just superior to the confluence of the iliac veins. An axial contrast-enhanced computed tomography of the pelvis (2B) demonstrates the right and left common iliac arteries (yellow arrows), the inferior vena cava (green arrow), and a filling defect (red arrow), within the confluence of the inferior vena cava with extension into the proximal right common iliac vein.

Multiple contrast-enhanced axial computed tomography (CT) images of the patient’s abdomen and pelvis, prior to initial thrombolysis intervention, were performed to assess the extent of thrombus at the venous confluence (Figure 2).

**Figure 3:**
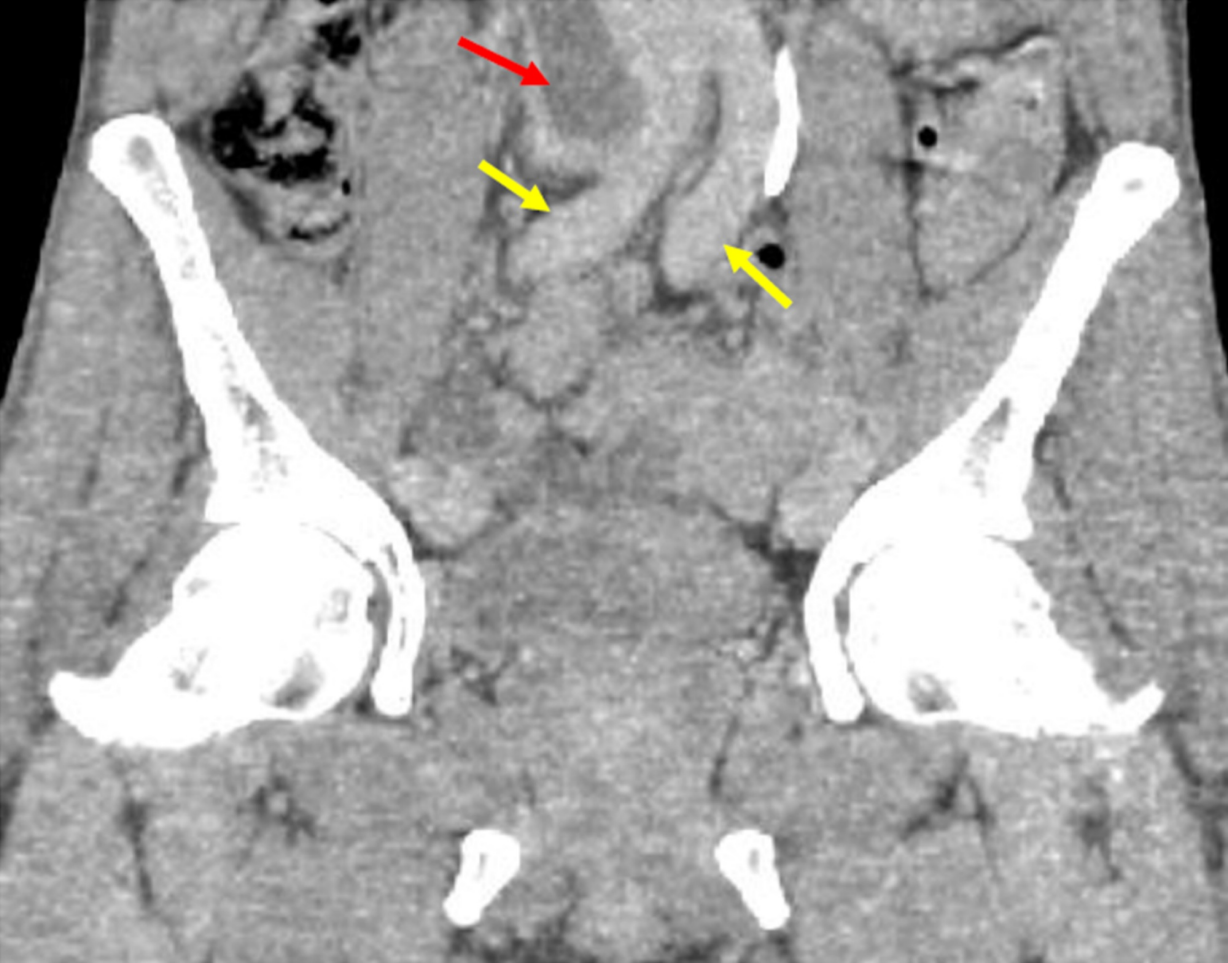
Coronal Computed Tomography Demonstrating Filling Defects within the Confluence of the Inferior Vena Cava Coronal computed tomography images of the abdomen and pelvis demonstrate the bilateral common iliac arteries (yellow arrows) and filling defect (red arrow) within the confluence of the inferior vena cava, consistent with a large thrombus.

Additional coronal CT imaging shows the venous thrombus adjacent to the aortic confluence (Figure 3).

An AngioJet® (Boston Scientific Corporation, Marlborough, Massachusetts, US) mechanical thrombectomy was attempted with an eight French catheter, through the right and left groin sheaths. A follow-up venogram demonstrated no significant improvement in thrombus burden. Suction catheter thrombectomy was then performed with a Penumbra® vacuum catheter (Penumbra Incorporated, Alameda, California, US) but showed no improvement following venogram. Following this, EKOS catheters were placed at the IVC confluence overnight, which also showed no improvement during post venogram. At this point, it was decided the iliocaval reconstruction with simultaneous removal of the IVC filter was necessary.

Iliocaval reconstruction and removal of the IVC filter

Initially, two Perclose ProGlide ® (Abbott Laboratories, Chicago, Illinois, US) sutures were placed at the ten and two o-clock positions of the bilateral groin sheaths. Under fluoroscopic guidance and over a Lundquist wire, the existing sheaths were upsized to 18 French dry seal sheaths. The sheaths were positioned in the IVC.

Through the left common femoral vein, an 18 French groin sheath was advanced over a guidewire, and the main body of a 26 mm by 12 mm by 12 cm Gore Excluder® AAA (W.L. Gore and Associates, Incorporated, Newark, Delaware, US) modular stent graft was advanced into the infrarenal vena cava.

**Figure 4:**
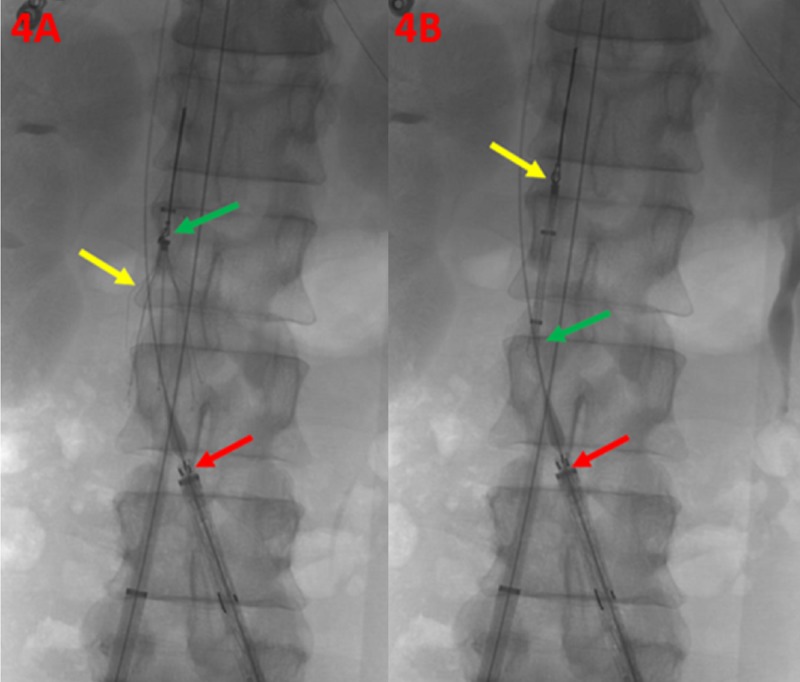
Retrieval of the Inferior Vena Cava Filter The infrarenal inferior vena cava filter is shown (4A) with a loop-snare retrieval device in place (green arrow), and the retrievable inferior vena cava filter in appropriate position without significant tilting (yellow arrow). The main body of the modular stent graft is noted within the proximal aspect of the left iliac vein (red arrow). The infrarenal inferior vena cava filter is shown (4B) captured (yellow arrow) within the retrieval sheath (green arrow). The main body of the modular stent graft is noted within the proximal aspect of the left iliac vein (red arrow).

In order to minimize the risk of thrombus embolization, excluding thrombus burden prior to removal of the IVC filter is crucial. Access was then obtained in the internal jugular vein of the patient with ultrasound guidance, and a guidewire was introduced into the IVC. Over the guidewire, a flush catheter was introduced into the IVC, and a venogram was performed. The existing removable IVC filter demonstrated no significant tilting, and a gooseneck loop-snare retrieval device/sheath was introduced into the IVC. The IVC filter was removed without incident (Figure 4).

**Figure 5:**
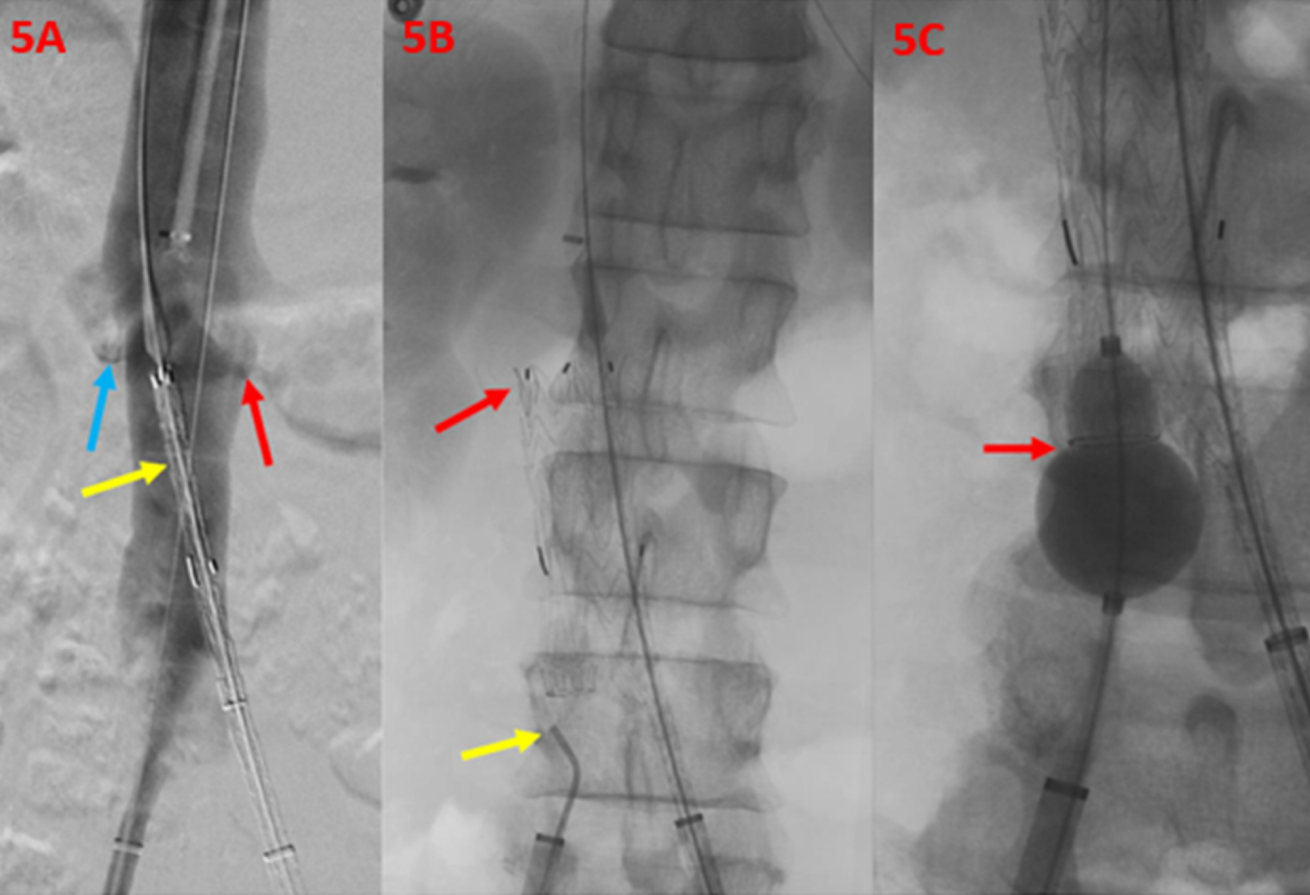
Deployment of the Aortic Endograft within the Confluence of the Inferior Vena Cava A flush venogram of the inferior vena cava (5A) demonstrates the right (blue arrow) and left (red arrow) renal veins. The main body of the modular stent graft is noted just inferior to the renal vein orifices (yellow arrow). Post-deployment imaging (5B) demonstrates the main body of the deployed modular stent graft (red arrow) and an additional catheter tip, within the right common iliac vein (yellow arrow). The venogram (5C) shows a balloon angioplasty (red arrow) used for confirmation of the contralateral limb wire catheterization.

Post-removal venography demonstrated no evidence of IVC injury or extravasation. The main body of the modular stent graft was then deployed with the proximal aspect marginally below the level of the renal veins. The 23 mm by 10 cm iliac limb was then appropriately positioned and deployed within the left common iliac vein (Figure 5).

**Figure 6:**
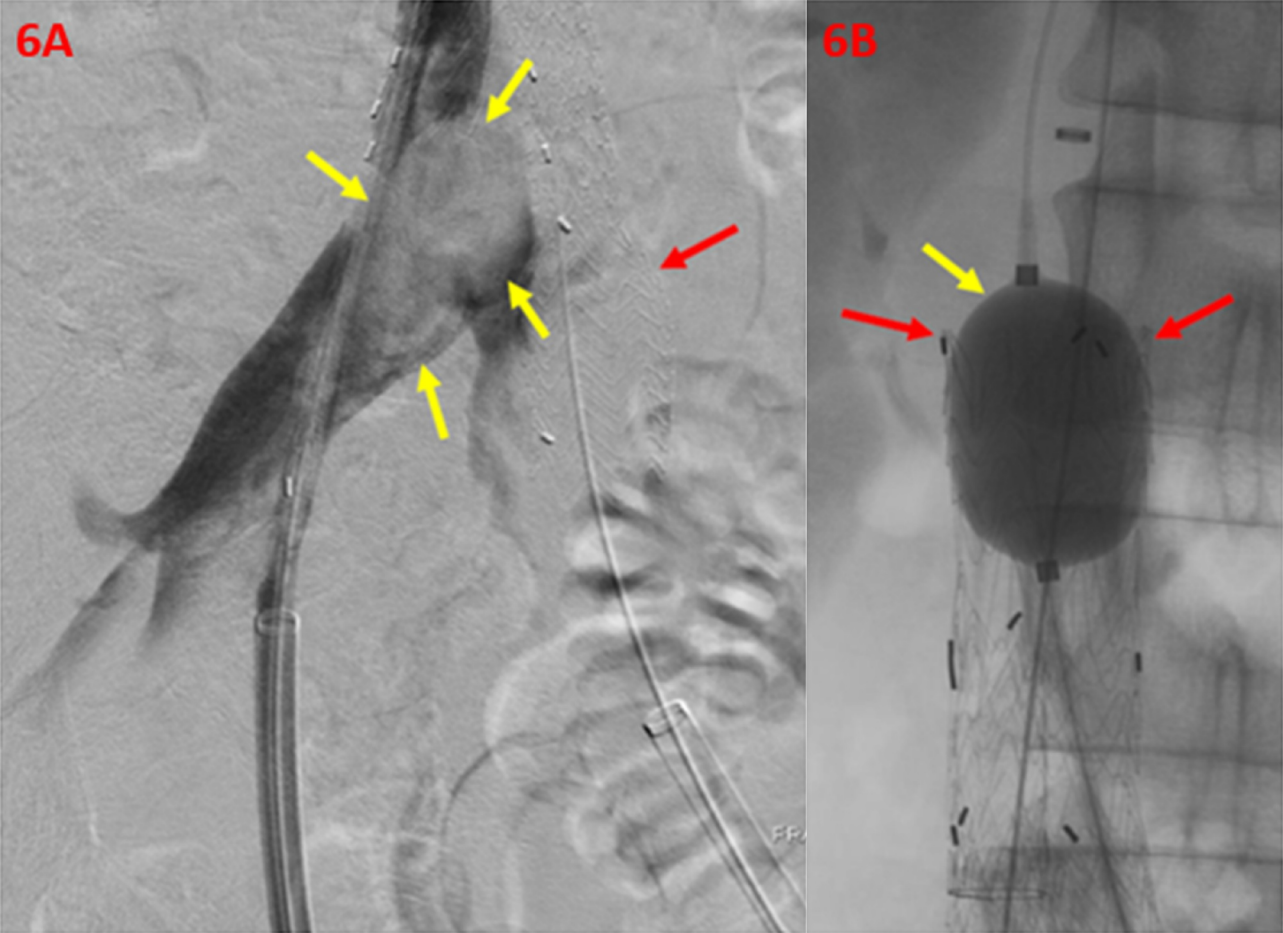
Correction of Persistent Filling Defect within the Right Iliac Vein and Confluence of the Inferior Vena Cava A right iliac vein angiogram (6A) demonstrates persistent filling defect (yellow arrows), representing thrombus burden within the right iliac vein and confluence of the inferior vena cava. The left limb of the stent graft is shown (red arrow) after successful ipsilateral deployment. A balloon angioplasty (6B) and expansion (yellow arrow) of the main body stent graft (red arrows) demonstrates appropriate wall apposition.

An additional 16 mm by 7 cm right iliac extender limb was then used for extension within the right iliac vein. Balloon venoplasty with a 32 mm Coda® (Cook Medical, Bloomington, Indiana, US) balloon catheter was then performed through the stent graft (Figure 6).

**Figure 7:**
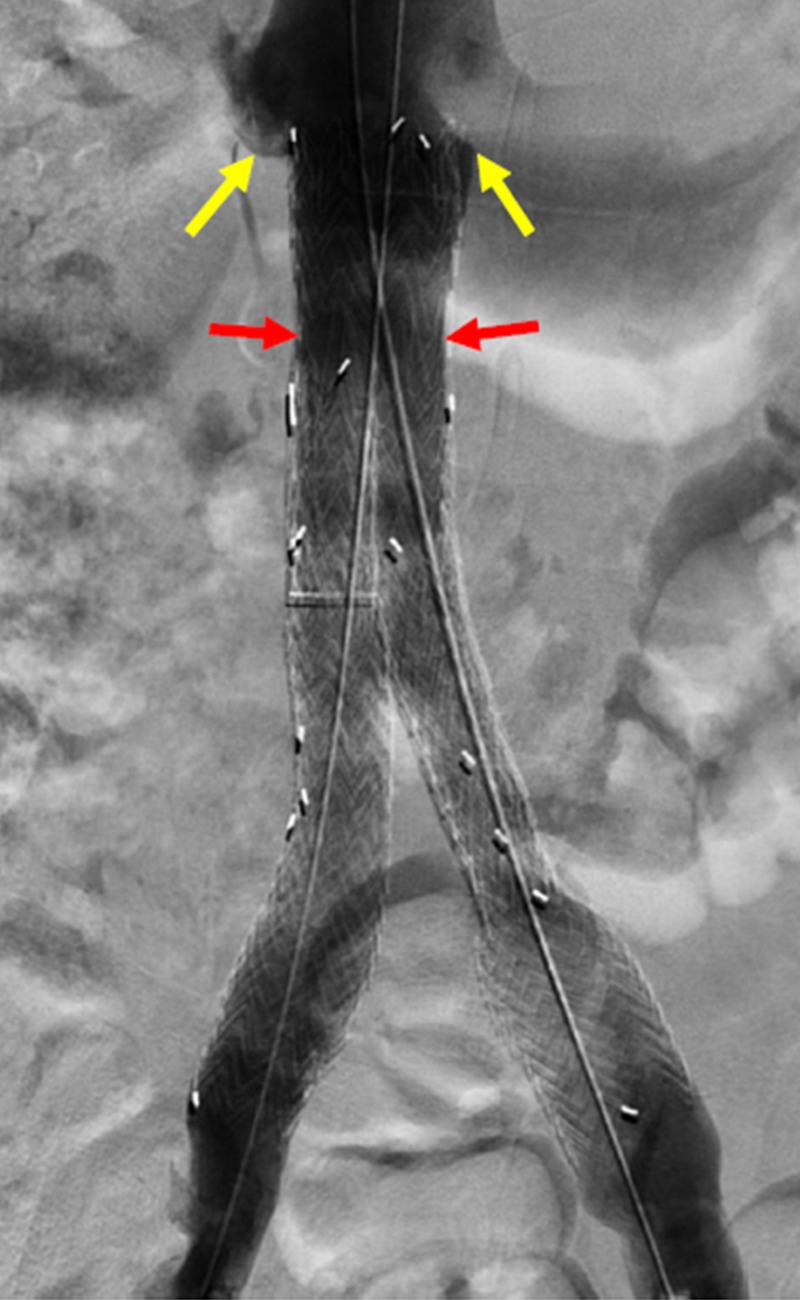
Successful Exclusion of the Large Thrombus Burden within the Iliocaval Confluence A final venogram of the inferior vena cava shows the main body stent graft at the level of the renal veins (yellow arrows). The right and left limbs of the excluder device are shown in the appropriate position with patency and absence of filling defect in the region of the confluence of the inferior vena cava (red arrows). This demonstrates successful exclusion of the large thrombus burden.

A follow-up venogram demonstrated patency and appropriate positioning of the stent graft, with exclusion of the previously seen thrombus at the iliocaval confluence. In addition, adequate inflow from both bilateral renal and internal iliac veins were noted on the final venogram (Figure 7).

All wires and catheters were removed. The patient’s groin accesses were closed using Perclose ProGlide® devices. Hemostasis was achieved, and sterile dressings were applied. The patient was transferred to the intensive care unit in stable condition; there were not any immediate post-procedural complications.

Post-procedural follow-up

**Figure 8:**
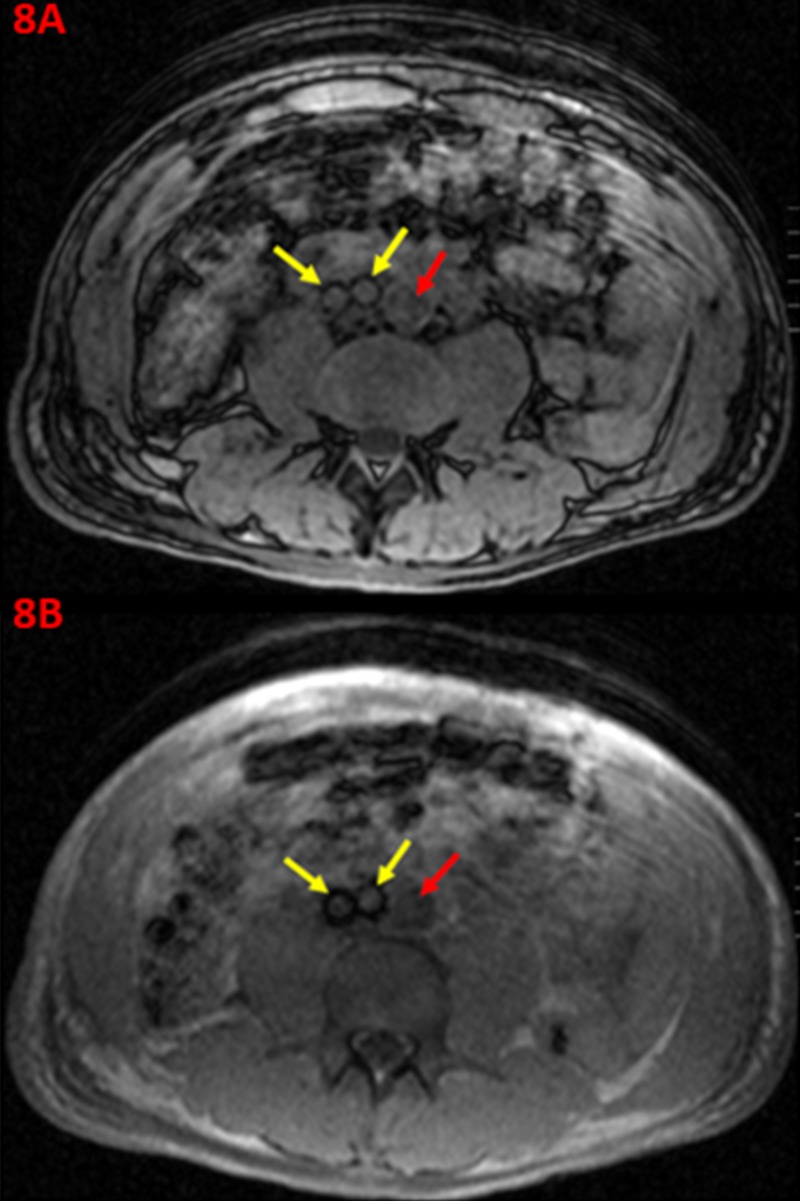
One-week Post-operative T1 Magnetic Resonance Images Displaying Stent Graft Patency within the Iliocaval Confluence A one-week post-operative axial T1 in/out of phase magnetic resonance image of the lower abdomen (8A) demonstrates patency of the right and left iliac veins (yellow arrows) and normal signal flow through the distal abdominal aorta (red arrow). An additional axial T1 in/out of phase magnetic resonance image of the lower abdomen at a slightly inferior axial cut (8B) demonstrates patency of the bilateral iliac veins (yellow arrows) with stent grafts visualized, and normal signal flow through the aorta (red arrow).

At the one-week post-operative follow-up visit, all the patient's symptoms resulting from venous dysfunction had improved. A magnetic resonance imaging (MRI) of the patient's abdomen and pelvis was performed in a 1.5 Tesla closed bore magnet, to assess for stent patency. These multi-sequence images demonstrate persistent patency of the bilateral iliac veins and confluence of the IVC on axial T1 in/out phase imaging (Figure 8).

**Figure 9:**
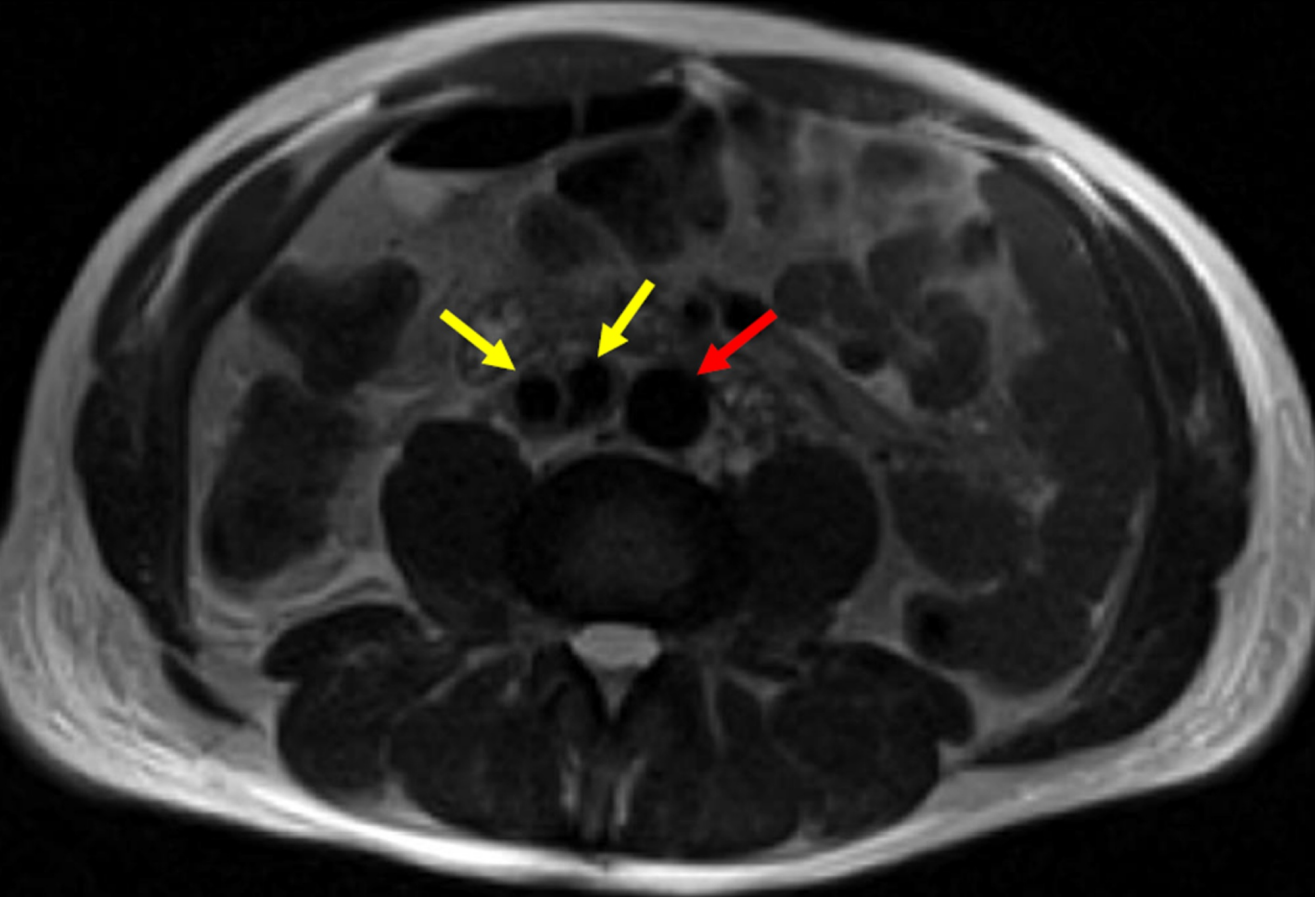
One-week Post-operative T2 Magnetic Resonance Image Displaying Stent Graft Patency within the Iliocaval Confluence A one-week post-operative axial T2 magnetic resonance image of the lower abdomen demonstrates low signal void in the bilateral iliac veins with patency through the stent graft (yellow arrows), and low signal flow void within the distal abdominal aorta (red arrow), consistent with patency.

An axial MRI T2-weighted image further shows patency of the aortic endograft placed one-week earlier at the iliocaval confluence (Figure 9).

**Figure 10:**
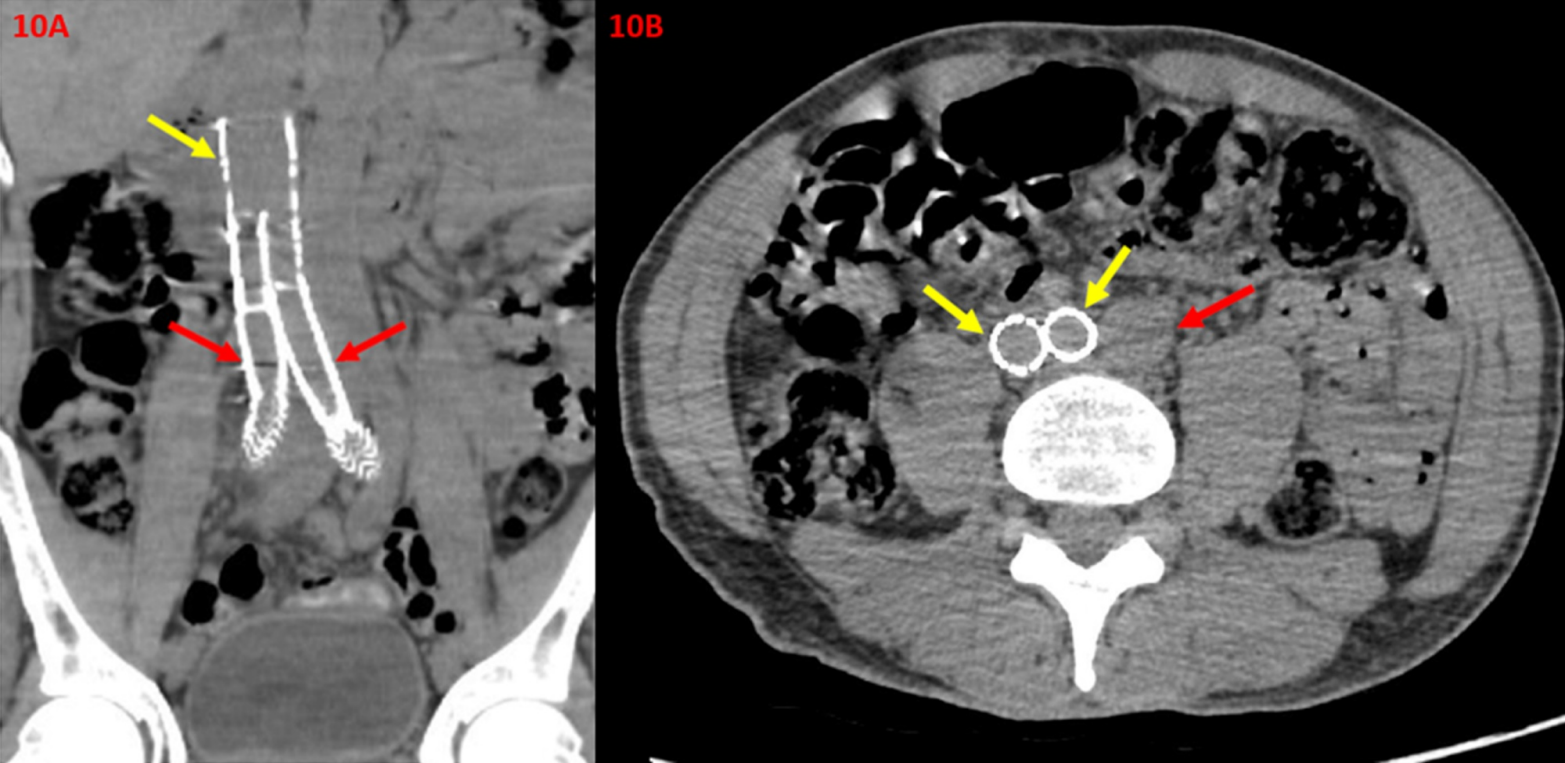
One-month Post-operative Non-contrast Enhanced Computed Tomography Images Displaying Stent Graft Patency within the Iliocaval Confluence A one-month post-operative non-contrast enhance coronal computed tomography of the abdomen and pelvis (10A) demonstrates patency within the iliocaval confluence (yellow arrow) and right and left iliac veins (red arrows). There is no evidence of thrombus, significant stenosis, or graft failure. A one-month post-operative non-contrast enhance axial computed tomography of the abdomen and pelvis (10B) demonstrates patency of the right and left iliac veins (yellow arrows) with no evidence of thrombus. Additionally, the unremarkable aorta (red arrow) demonstrates patency with no evidence of aneurysmal dilation or abnormality.

Later at the one-month post-operative follow-up, a non-contrast enhanced CT of the abdomen and pelvis demonstrates continued patency within the iliocaval confluence (Figure 10).

In all follow-up periods, there was no evidence of stent graft migration, stenosis, or thrombus within the iliac venous system or the confluence and proximal IVC. Additionally, the regional distal abdominal aorta is noted to be unremarkable.

As expected, due to balloon angioplasty of the main body modulator stent graft and the precise placement of the bilateral iliac limb excluder stents, no evidence of stent failure or migration was visualized. These multimodal follow-up cross-sectional images further demonstrate the success of the iliocaval reconstruction in this patient.

After the one-month follow-up period, the patient’s symptoms continued to improve, and he was placed on anticoagulation management. Currently, the plan is to follow-up with patient in six months.

## Discussion

Multiple published studies have reported favorable prognoses for patients who receive venoplasty and stent placement, in an attempt to achieve iliocaval patency. These studies report a technical success rate of thrombotic iliocaval reconstruction that ranges from 83%-95% [11]. In a retrospective study of 120 patients with inferior vena cava filter related thrombosis, the technical success rate of iliocaval reconstruction was 100% [11]. Another European retrospective study of 89 patients demonstrated primary, assisted-primary, and secondary iliocaval patency rates of 83%, 89%, and 93%, respectively, at three and ten years [1]. Furthermore, Neglan et al. compared patency of iliocaval reconstructions between cohorts of patients with and without inferior vena cava filters, and found that the presence of an inferior vena cava filter did not impact long-term patency [12]. This study showed that the only factor associated with lower patency rate was the severity of initial thrombotic disease. Therefore, we can infer that the prognosis for post-procedural iliocaval patency in this patient, exclusively relies on the severity of their subacute iliocaval occlusion. In this case, it may seem that the patient will have an unfavorable prognosis in the post-procedural period; however, the entirety of the thrombus burden was addressed completely by the placement of the aortic endograft.

Very few complications have been reported after stent placement during iliocaval reconstruction, the most significant being renal vein thrombosis, which occurred in one out of 120 patients in the Chick et al. study [11]. The most common complication is self-limited access site hematoma, which can be managed with manual compression or assisted pressure devices [1]. Rare cases of venous perforation or arterial injury can be hemodynamically significant, and may require additional imaging with subsequent endovascular management.

Clinical evaluation for iliocaval insufficiency, secondary to acute or chronic thrombotic disease, should be performed first to consider if a patient is in need of endovenous intervention. Clinical evaluation for iliocaval disease includes a focused patient history, including symptom onset, functional impairment related to venous occlusion, comorbidities, history of anticoagulation, and review of prior procedures that may affect prospective endovenous intervention. Therefore, imaging is also essential for pre-procedural planning and diagnosis. Ultrasonography may be used to demonstrate femoral and peripheral lower extremity thrombosis, while computed tomography venography or magnetic resonance venography may be used to demonstrate central iliocaval thrombosis [1]. Once the appropriate clinical evaluation and diagnostic imaging have been obtained, endovenous intervention can be considered.

The current indications for performing endovenous iliocaval reconstruction include chronic iliocaval occlusion with recurrent deep vein thrombosis, severe post-thrombotic syndrome, or related symptoms that restrict normal activity or quality of life [1]. Ideally, endovenous iliocaval reconstruction should be performed within two weeks of onset in the acute setting, or after four months in the case of chronic occlusion [13]. This time frame offers any existing thrombosis the opportunity to adhere to the venous lumen, allowing for more accessible recanalization and reconstruction if endovenous stent placement is required. In this case of subacute iliocaval occlusion, the size of the residual thrombus burden was too great for an uncovered bare metal stent to be used. Using uncovered stents within the iliocaval confluence would also increase the likelihood for contralateral iliac vein and post-procedural deep vein thrombosis to occur [9]. Furthermore, a covered aortic stent graft was used to reduce the risk of excluded thrombus penetration through the stent interstices, as would be the case in an uncovered stent.

Though clinical and diagnostic work-up can guide the appropriate interventional management of chronic venous insufficiency, in the setting of acute-on-chronic venous disease, as in this case, can be unpredictable for an interventional operator. In this setting, the interventional operator must be willing to use devices that are not designed for certain interventions, and adapt their technical knowledge to solve realistically possible situations. In this case, the patient benefited from the first documented placement of an aortic endograft within the iliocaval confluence, to resolve both extensive and technically challenging venous disease. The situation was, of course, not ideal or certain for success; however, the result was the resolution of the patient's subacute iliocaval occlusive disease. As shown in the results, the interventional procedure leads to iliocaval patency and the absence of post-procedural complications. Inevitably, as demonstrated in the preceding case, interventional management must continue to evolve and adapt with the progressive presentation of venous disease.

## Conclusions

Interventional management has propelled the treatment for venous dysfunction and the sequalae for acute and chronic iliocaval disease forward, as it is an efficient and minimally invasive means for treatment. The limitations of readily available devices may be overcome by the technical skill of an experienced interventional operator, and the insight to adapt them for purposes never used before. An example of this is the reconstruction of an anatomically challenging location, such as the iliocaval confluence in this patient with extensive thrombus burden, with a covered aortic stent graft.
